# A pooled analysis of on-the-road highway driving studies in actual traffic measuring standard deviation of lateral position (i.e., “weaving”) while driving at a blood alcohol concentration of 0.5 g/L

**DOI:** 10.1007/s00213-016-4519-z

**Published:** 2017-01-09

**Authors:** S. Jongen, A. Vermeeren, N. N. J. J. M. van der Sluiszen, M. B. Schumacher, E. L. Theunissen, K. P. C. Kuypers, E. F. P. M. Vuurman, J. G. Ramaekers

**Affiliations:** 1 Department of Neuropsychology and Psychopharmacology, Faculty of Psychology and Neuroscience, Maastricht University, Universiteitssingel 40, 6200 MD Maastricht, The Netherlands; 2 Federal Highway Research Institute (BASt), Section U3/Traffic Psychology, Traffic Education, Bruederstrasse 53, D-51427 Bergisch Gladbach, Germany

**Keywords:** Alcohol, On-the-road driving, Standard deviation of lateral position, Clinical relevance

## Abstract

**Introduction:**

The on-the-road highway driving test is generally regarded as a gold standard for assessing drug-induced driving impairment. The primary outcome measure is the standard deviation of lateral position (SDLP), a measure of road tracking error or “weaving”. The test has been calibrated for incremental doses of alcohol almost 30 years ago in order to define the impact of drug-induced impairment in terms of blood alcohol concentration (BAC) equivalents. Drug-induced changes in SDLP exceeding 2.4 cm have been evaluated as clinically relevant ever since. The present analysis was conducted to assess the robustness of the alcohol effect in a range of on-the-road driving studies which have been conducted since the initial alcohol calibration study.

**Methods:**

The present study pooled data of 182 participants from nine placebo-controlled crossover studies who performed the highway driving test, while their BAC was at or just below the legal limit for drivers (i.e., 0.5 g/L).

**Results:**

Overall, mean SDLP increased with 2.5 cm (95% CI 2.0–2.9 cm). Equivalence testing showed that the clinical relevance criterion value of 2.4 cm fell well within the 95% CI in each individual study. Gender did not affect alcohol-induced changes in SDLP.

**Discussion:**

These results demonstrate the robustness and validity of the clinical relevance criterion for SDLP as measured during on-the-road driving.

## Introduction

Evaluation of medicinal drug effects on the ability to operate a motor vehicle is strongly recommended to inform both users and prescribers (Food Drug Administration 2015; Kay and Logan 2011). The highway driving test in actual traffic is generally considered as an experimental gold standard to assess drug-induced driving impairment. The primary outcome measure of the driving test is standard deviation of lateral position (SDLP) (O’Hanlon 1984). This standardized driving test has been applied in over 75 studies and demonstrated sensitivity to the impairing effects of several central nervous system (CNS) drugs (Brookhuis et al. 1990; Ramaekers 1998, 2003; Theunissen et al. 2014; Vermeeren 2004; Vermeeren et al. 2009; Verster et al. 2004).

A common approach to determine clinical relevance of drug-induced impairment is to compare their effects to that of a benchmark drug known to jeopardize traffic safety, such as alcohol (Walsh et al. 2008). Alcohol has been shown to exponentially increase crash risk with increasing blood alcohol concentrations (BAC) (Borkenstein 1974; Krüger et al. 1990), and legal per se limits for driving under the influence of alcohol have been implemented worldwide (Brookhuis et al. 2003). SDLP was one of the first standardized driving measures calibrated for incremental doses of alcohol (Louwerens et al. 1987). The results enabled researchers in subsequent studies to interpret the magnitude of drug-induced impairment in terms of BAC equivalents. In the original alcohol calibration study, participants conducted the driving test during five alcohol conditions with a mean BAC of 0, 0.3, 0.6, 0.9, and 1.2 g/L. The driving test was conducted on a 25-km closed course in which the participants had to maintain a constant speed of 90 km/h and a steady lateral position. Alcohol produced an exponential rise in SDLP with increasing BACs. Curve fitting was subsequently applied to define changes in SDLP as a function of BAC (Fig. 2). Increments in SDLP of 2.4, 4.2, and 5.1 cm were defined as clinically relevant cutoff points representing BACs of 0.5, 0.8, and 1.0 g/L, respectively (Verster and Ramaekers 2009).

In subsequent studies, the same driving test was used on a 100-km primary highway with a constant speed of 95 km/h. In these studies, the cut-off value of 2.4 cm was used as a criterion level to define clinically relevant driving impairment of drugs other than alcohol, as a BAC of 0.5 g/L is the legal limit for driving under the influence of alcohol in most countries. Nine of those studies also included alcohol treatment as a positive control or as additional treatment to study drug-alcohol interactions. In each study, an alcohol dosing regimen was used to achieve a BAC just below the legal limit for drivers, i.e., 0.5 g/L, at the start of the driving test. The present study pooled these datasets in order to evaluate the robustness of the alcohol-induced changes in SDLP at a BAC of 0.5 g/L over different settings. Changes of SDLP at a BAC of 0.5 g/L and the associated effect size observed in these nine studies were compared to those in the original alcohol calibration study. In addition, we performed a symmetry analysis to determine the risk of impaired driving (Laska et al. 2012). If alcohol does not increase the risk of impaired driving performance, the changes in SDLP (i.e., alcohol minus placebo) will be random and symmetrical around zero. If the symmetry analysis shows significantly more subjects with changes above the threshold of +2.4 cm compared to the mirrored threshold of −2.4 cm, it can be concluded that alcohol does increase the risk of impaired driving performance.

## Methods

### Studies

Table 1 shows a summary of study characteristics. Driving data of placebo and alcohol treatments from nine studies conducted by Maastricht University were included (Kuypers et al. 2006; Ramaekers et al. 2000; Ramaekers et al. 1992; Schumacher et al. 2011; Schumacher 2014; van der Sluiszen et al. 2016; Vermeeren and O’Hanlon 1998; Vermeeren et al. 2002a; Vermeeren et al. 2002b). Only studies which aimed to reach a BAC of 0.5 g/L at the start of the driving test were included. Two studies aimed to reach a BAC of 0.35 g/L (Vuurman et al. 1996) and 0.8 g/L (Riedel et al. 1987), respectively, and were therefore excluded. All studies were conducted according to a balanced, single or double-blind, placebo-controlled crossover design, including one treatment condition consisting of the administration of alcohol aiming to reach a BAC just below 0.5 g/L at the start of the driving test.

**Table 1:** Summary of nine studies included in the pooled analysis. All studies were conducted following a double-blind crossover design

Study	Sample size	Age range (y)	Alcohol dosing	Time of dosing until start driving test	Time of testing	BAC start driving-BAC end driving (in g/L)
Ramaekers et al. 1992	All 16^a^	22–35	0.72 g/kg	3 h	Noon	0.37–0.20
Vermeeren and O’Hanlon 1998	All 24 Male 12 Female 12	22–44	7 constant 5.6 g, a 10 g, and 2 adjustable doses	3 h	Noon	0.45–0.37
Ramaekers et al. 2000	All 18^a^	20–28	0.60 g/kg	1.5 h	Evening	0.50–0.35
Vermeeren et al. 2002a	All 19 Male 9 Female 10	21–45	Males 0.43 g/kg Females 0.36 g/kg	1 h	Morning	0.42–0.23
Vermeeren et al. 2002b	All 30 Male15 Female 15	21–45	Males 0.43 g/kg Females 0.36 g/kg	2.15 h	Noon	0.37–0.24
Kuypers et al. 2006	All 18 Male 9 Female 9	20–37	0.70 g/kg	2 h	Noon	0.37–0.29
van der Sluiszen et al. 2016	All 25 Male 12 Female 13	21–45	Males 3 doses (0.23, 0.14, 0.14 g/kg) Females 3 doses (0.21, 0.13, 0.13 g/kg)	1 h	Noon	0.45–0.30
Schumacher et al. 2011	All 17 Male 12 Female 5	23–58	Males 3 doses (0.23, 0.14, 0.14 g/kg) Females 3 doses (0.21, 0.13, 0.13 g/kg)	1 h	Morning	0.49–0.39
Schumacher 2014	All 15 Male 6 Female 9	23–59	Males 3 doses (0.23, 0.14, 0.14 g/kg) Females 3 doses (0.21, 0.13, 0.13 g/kg)	1 h	Morning	0.50–0.35

*BAC* blood alcohol concentration

^a^No gender data available

### Participants

The complete dataset included 182 volunteers (92 males, 90 females) in the age range of 21 to 59 years. All participants were healthy volunteers as determined by a medical history questionnaire and physical examination, including electrocardiogram, blood hematology and chemistry, and urinalysis. Common inclusion criteria were possession of a valid driving license for 3 years or more, driving experience of at least 3000 km per year in the past 3 years, and a body mass index between 19 and 29 kg m^−2^. Exclusion criteria were clinically significant physical or mental disorders; drug abuse; use of systematic medication except oral contraceptives; excessive use of caffeine (>6 beverages containing caffeine per day), alcohol (>21 alcohol-containing beverages per week), and smoking (>6 cigarettes per day).

All studies were conducted at Maastricht University in accordance with the code of ethics on human experimentation established by the Declaration of Helsinki (1964) and its subsequent amendments. Studies were approved by the medical ethics committee of Maastricht University and University Hospital of Maastricht. Participants signed an informed consent form before initiation of any study-related assessment.

### Alcohol administration

**Fig. 1:**
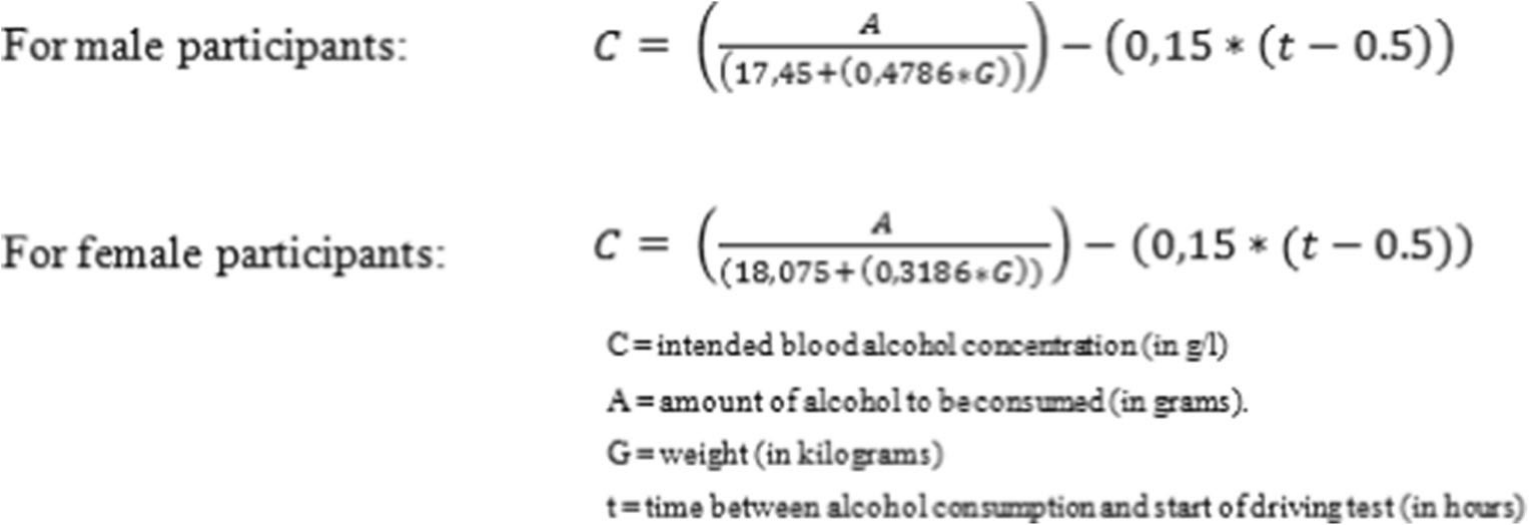
Formulas for calculating blood alcohol concentrations

All studies used weight-calibrated doses of pure alcohol (99.8%) mixed with orange juice to achieve a BAC just under the legal limit for drivers (i.e., 0.5 g/L) at the start of the driving test. Alcohol-dosing regimens were either single doses or multiple titrated doses. In four studies (Kuypers et al. 2006; Ramaekers et al. 1992, 2000; Vermeeren and O’Hanlon 1998), gender differences were not taken into account for calculating the dose. In five studies (Schumacher et al. 2011; Schumacher 2014; van der Sluiszen et al. 2016; Vermeeren et al. 2002a; Vermeeren et al. 2002b), the dose was calculated using the improved version (Watson 1981) of the Widmark formula (Widmark 1932; Fig. 1). Breath samples were obtained at the start and end of the driving test using a Lion SD-3, Lion SD-400 (Lion Laboratories Ltd., Barry, UK), or a Dräger Alcotest 6510. In all studies BACs declined over time during driving (Table 1).

### Placebo conditions

Driving performance during placebo treatment served as a reference for determining alcohol-induced changes in SDLP. In four studies, a placebo condition without alcohol administration was identified (Ramaekers et al. 1992; van der Sluiszen et al. 2016; Vermeeren and O’Hanlon 1998; Vermeeren et al. 2002a). In two studies (Ramaekers et al. 2000; Vermeeren et al. 2002b), alcohol placebo drinks consisted of a glass of orange juice flavored with Grand Marnier essence. In three studies (Kuypers et al. 2006; Schumacher et al. 2011; Schumacher 2014), a small amount (3 ml) of alcohol floating on the surface of a glass of orange juice was used to pretend that the beverage contained alcohol.

### Highway driving test

**Fig. 2:**
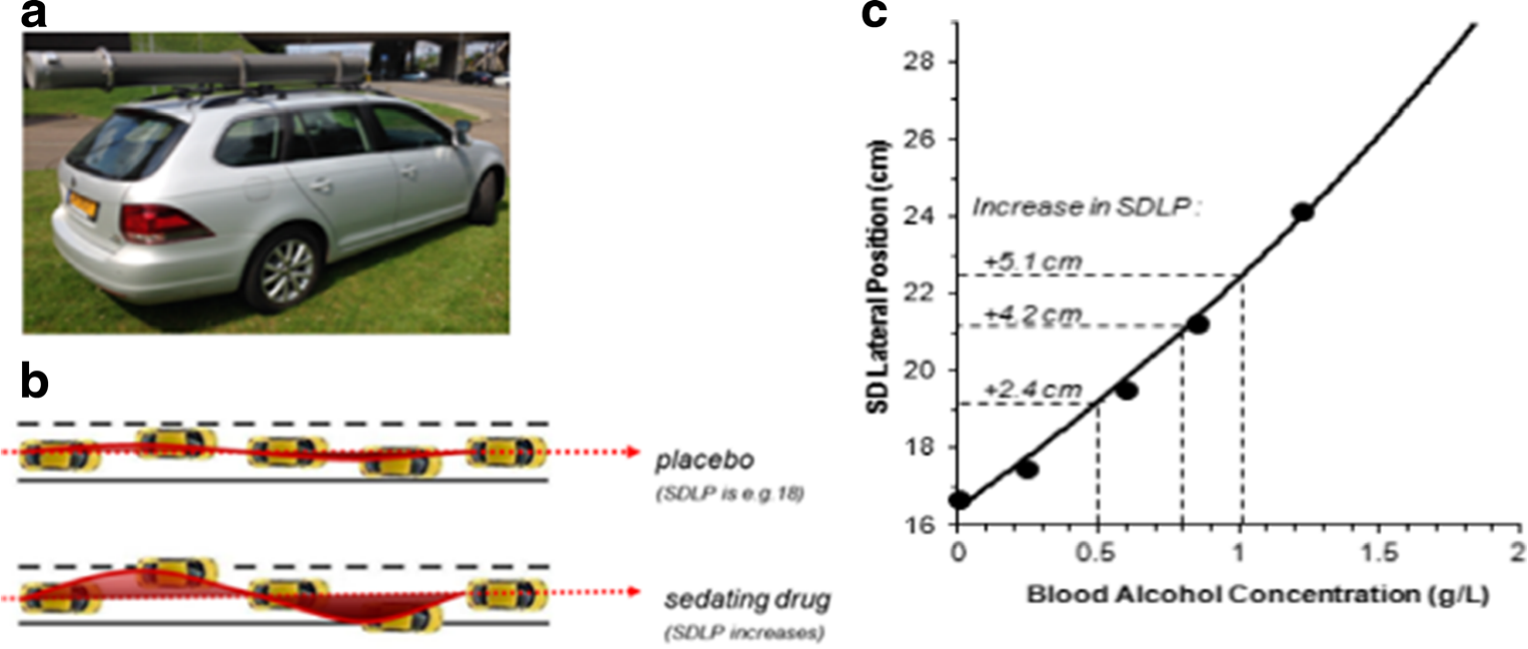
Standardized highway driving test. **a** Participants drive a specially instrumented vehicle for about 1 h over a 100 km primary highway circuit, accompanied by a licensed driving instructor having access to dual controls. The participants’ task is to drive with a steady lateral position between the delineated boundaries of the slower right traffic lane, while maintaining a constant speed of 95 km/h. **b** The standard deviation of lateral position (SDLP in centimeter) is an index of road tracking error or “weaving”. **c** The relationship between blood alcohol concentration and SDLP as obtained by Louwerens et al. (1987)

In the standardized on-the-road highway driving test (O’Hanlon 1984), the participant operates a specially instrumented vehicle over a 100-km primary highway circuit in actual traffic, accompanied by a licensed driving instructor having access to dual controls. The task of the participant is to maintain a constant speed of 95 km/h and a steady lateral position between the delineated boundaries of the right traffic lane. The vehicle speed and lateral position are recorded continuously. These signals are digitized at a rate of 4 Hz and edited offline to remove data recorded during overtaking maneuvers or disturbances caused by roadway or traffic situations. The remaining data are then used to calculate mean values and standard deviation of lateral position (SDLP, in centimeter) for each successive 5-km segment and, as the square root of pooled variance over all segments, for the test as a whole. SDLP is the primary performance parameter, which is an index of road-tracking error or “weaving” (Fig. 2). Several different cars and circuits in Belgium and the Netherlands have been used over the years.

### Statistical analysis

The overall alcohol effect was analyzed using a 2 × 2 × 9 general linear model (GLM) repeated measures analysis with alcohol treatment (placebo and alcohol) as within-subject factor and gender and study as between-subject factors. In addition, alcohol and gender effects were assessed for each study separately by using 2 × 2 GLM repeated measures. Equivalence testing was applied to assess whether the predefined alcohol criterion of 2.4 cm fell within the 95% confidence interval (CI) of the mean difference scores (i.e., ΔSDLP) of the individual studies.

To determine the magnitude of the alcohol effect on SDLP, effect sizes (ES) for repeated measures designs were calculated for all studies combined and for each study separately (ES = t_c_[2(1-r)/n]^1/2^) (Dunlap et al. 1996). An ES between 0.00 and 0.19 was considered small, between 0.20 and 0.69 moderate, and higher than 0.70 large (Lakens 2013).

To detect an asymmetry in the distribution of the individual difference scores between SDLP after alcohol and placebo, a McNemar test was used (Laska et al. 2012). This test examines the difference in proportions of impaired and improved drivers following alcohol using a generalized sign test over the relevant threshold of 2.4 cm, which is the predefined criterion used for a mean increase of SDLP. Symmetry implies that the probability of impairment over placebo is the same as the probability of improvement. Rejecting the null hypothesis implies that the two probabilities are unequal, indicating that alcohol does increase the risk of impaired driving performance.

## Results

### Missing data

Gender data could not be retrieved in two studies (Ramaekers et al. 1992, 2000). These datasets were included in the overall effect of alcohol on SDLP, but were removed from gender analyses.

### Mean changes in SDLP scores

Table 2 shows overall and individual study means of SDLP scores in placebo and alcohol conditions and their corresponding mean (95% CI) ΔSDLP. Repeated measures analysis of variance showed that the overall mean increase (95% CI) in SDLP was 2.5 cm (2.0–2.9) (*F*_1, 181_ = 132.78, *p* < 0.001) in the alcohol condition compared to placebo. The overall effect size was moderate 0.54 (range 0.45–0.73). Overall, alcohol-induced changes in SDLP did not significantly differ between studies (*F*_6,134_ = 1.30, *p* = 0.263) and gender (*F*_1,134_ = 0.14, *p* = 0.708).

**Table 2:** Overall mean (SD) score of standard deviation of lateral position (SDLP) after placebo (PBO) and alcohol (ALC), mean (95% CI) change scores (Δ SDLP), repeated measure analyses of variance, Dunlap’s effect sizes and proportion improved vs. impaired driver, and for each study and gender separately

Study		SDLP PBO (SD)	SDLP ALC (SD)	ΔSDLP (95% CI)	F	*p*	Dunlap’s ES	Improved/impaired
Ramaekers et al. 1992	All^a^	20.2 (4.0)	23.2 (4.6)	3.0 (0.9–5.2)	8.9	<0.01	0.69	00/08
Vermeeren and O’Hanlon 1998	All	21.9 (5.7)	25.5 (7.3)	3.6 (1.9–5.3)	19.0	<0.001	0.52	02/15
	Male	22.2 (5.6)	25.3 (7.1)	3.1 (−0.3–6.4)	–	NS	0.47	02/06
	Female	21.6 (6.1)	25.8 (7.8)	4.1 (2.6–5.7)	34.5	<0.001	0.42	00/09
Ramaekers et al. 2000	All^a^	22.3 (4.8)	24.4 (3.4)	2.0 (0.5–3.5)	8.3	<0.05	0.45	02/08
Vermeeren et al. 2002a	All	20.0 (3.6)	22.3 (3.9)	2.3 (1.5–3.1)	37.3	<0.001	0.59	00/10
	Male	19.5 (4.4)	21.1 (4.4)	1.6 (0.4–2.9)	8.9	<0.05	0.37	00/03
	Female	20.5 (2.8)	23.5 (3.3)	3.0 (1.9–4.0)	39.5	<0.001	0.93	00/07
Vermeeren et al. 2002b	All	17.7 (3.0)	19.4 (3.7)	1.7 (0.7–2.6)	12.9	<0.01	0.49	00/07
	Male	18.0 (3.2)	20.3 (4.2)	2.3 (0.3–4.2)	6.4	<0.05	0.58	00/06
	Female	17.4 (2.7)	18.5 (2.8)	1.1 (0.6–1.5)	24.3	<0.001	0.40	00/01
Kuypers et al. 2006	All	20.6 (3.9)	23.5 (4.0)	2.9 (1.2–4.5)	13.5	<0.01	0.73	00/09
	Male	20.2 (3.9)	23.8 (3.8)	3.6 (1.1–6.0)	11.2	<0.05	0.92	00/05
	Female	21.1 (4.0)	23.3 (4.4)	2.2 (−0.5–4.8)	–	NS	0.51	00/04
van der Sluiszen et al. 2016	All	17.0 (2.6)	19.4 (3.4)	2.5 (1.7–3.2)	46.7	<0.001	0.72	00/12
	Male	17.2 (2.2)	18.9 (2.8)	1.7 (0.6–2.8)	11.5	<0.01	0.63	00/03
	Female	16.8 (3.1)	20.0 (4.0)	3.2 (2.2–4.1)	50.3	<0.001	0.79	00/09
Schumacher et al. 2011	All	18.3 (4.1)	20.7 (3.3)	2.4 (1.2–3.6)	18.4	<0.05	0.61	00/10
	Male	18.5 (4.5)	20.5 (3.6)	2.0 (0.5–3.6)	8.1	<0.05	0.47	00/06
	Female	17.9 (3.4)	21.3 (2.7)	3.4 (1.1–5.8)	16.4	<0.05	1.06	00/04
Schumacher 2014	All	19.7 (3.3)	21.5 (3.2)	1.9 (0.6–3.2)	10.1	<0.01	0.58	00/07
	Male	19.2 (5.2)	22.0 (3.9)	2.8 (0.3–5.2)	8.0	<0.05	0.52	00/03
	Female	20.0 (1.6)	21.3 (2.9)	1.3 (−0.4–3.0)	–	NS	0.48	00/04
Total	All 182	19.6 (4.3)	22.1 (4.8)	2.5 (2.0–2.9)	132.8	<0.001	0.54	04/86
	Male 75^b^	19.2 (4.3)	21.6 (4.8)	2.4 (1.7–3.1)	42.8	<0.001	0.51	02/32^b^
	Female 73^b^	19.2 (4.0)	21.7 (5.0)	2.5 (2.0–3.1)	93.1	<0.001	0.51	00/38^b^

*BAC* blood alcohol concentration, *PBO* placebo, *ALC* alcohol, *ES* effect size, *NS* not significant

^a^No gender data available

^b^Gender data deviates from total, because no gender data were available of 34 participants﻿

**Fig. 3:**
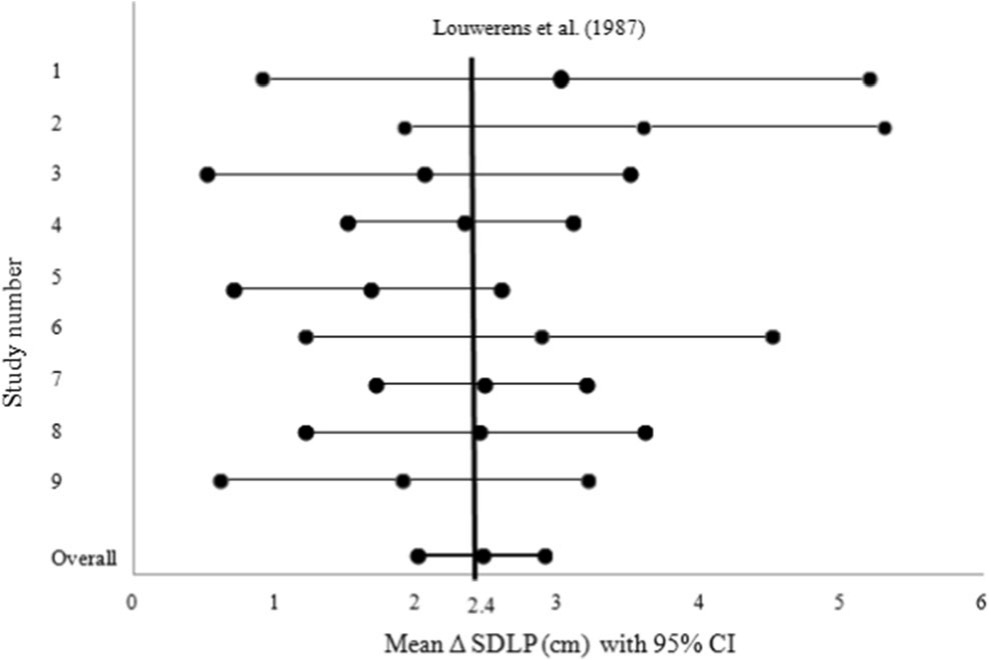
Overall mean change of standard deviation of lateral position (Δ SDLP, in centimeter) with 95% confidence interval (CI) and mean changes of SDLP with 95% CI of each individual study after alcohol reaching a blood alcohol concentration (BAC) of 0.5 g/L. The *vertical black line* is the clinically relevant cutoff point of 2.4 cm, as defined by Louwerens et al. (1987). Study 1: Ramaekers et al. 1992, Study 2: Vermeeren and O’Hanlon 1998, Study 3: Ramaekers et al. 2000, Study 4: Vermeeren et al. 2002a, Study 5: Vermeeren et al. 2002b, Study 6: Kuypers et al. 2006, Study 7: van der Sluiszen et al. 2016, Study 8: Schumacher et al. 2011, Study 9: Schumacher 2014

Alcohol-induced increments in SDLP ranged from +1.9 to +3.6 cm across all driving studies. Equivalence testing showed that in each individual study, the criterion value of 2.4 cm fell well within the 95% CI (Fig. 3). Overall, the mean increase of SDLP in males (95% CI) was 2.4 cm (1.7–3.1) with an effect size of 0.51 (range 0.37–0.92) and in females 2.5 cm (2.0–3.1) with an effect size of 0.51 (range 0.40–1.06). Effect sizes of individual studies are reported in Table 2.

### Proportion of impaired and improved drivers

**Fig. 4:**
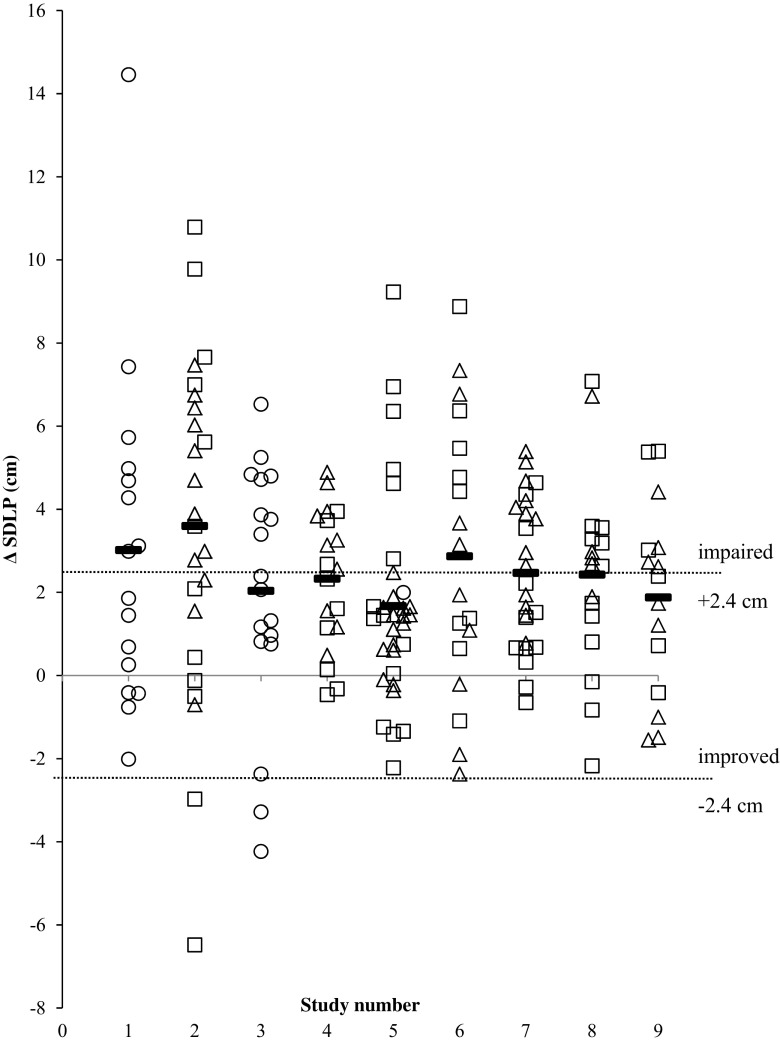
Individual and mean (*horizontal lines*) alcohol-placebo changes in driving performance as measured by the standard deviation of lateral position (SDLP). Change scores for each individual study are shown separately for males (*squares*) and females (*triangles*) and for individuals for whom gender data was missing (*circles*). *Dotted lines* show thresholds for impaired (changes above 2.4 cm) and improved driving (changes below −2.4 cm). Study 1: Ramaekers et al. 1992, Study 2: Vermeeren and O’Hanlon 1998, Study 3: Ramaekers et al. 2000, Study 4: Vermeeren et al. 2002a, Study 5: Vermeeren et al. 2002b, Study 6: Kuypers et al. 2006, Study 7: van der Sluiszen et al. 2016, Study 8: Schumacher et al. 2011, Study 9: Schumacher 2014

Symmetry analysis of SDLP changes in individual drivers confirmed that alcohol significantly impairs driving performance (McNemar test 72.90, *p* < 0.001). Overall, 47.3% (86 out of 182 participants) of the drivers showed increments in SDLP that exceeded the criterion level of 2.4 cm. In contrast, only 2.2% (i.e., 4 participants) of the drivers showed improvement following alcohol that exceeded the mirrored criterion value of −2.4 cm. In males, the distribution of individual drivers showing driving impairment or improvement beyond the criterion value was 42.7% (i.e., 32 participants) and 2.7% (i.e., 2 participants) (McNemar test 24.74, *p* < 0.001). In females, 52.1% (i.e., 38 participants) of the drivers showed alcohol-induced impairment, whereas none improved (McNemar test 36.03, *p* < 0.001) (Fig. 4). It should be noted that gender data deviates from the total regarding proportion of impaired and improved drivers, because no gender data were available for the 34 participants.

## Discussion

The aim of the present paper was to evaluate the robustness of an SDLP increase of 2.4 cm during highway driving at a BAC of 0.5 g/L, as determined by an alcohol calibration study almost 30 years ago (Louwerens et al. 1987). For this purpose, data from 182 participants of nine placebo-controlled studies using the same driving test and comparable methods were pooled and analyzed. It was found that alcohol at a BAC of 0.5 g/L led to a mean ΔSDLP of 2.5 cm, and that alcohol-induced changes in SDLP did not significantly differ between individual driving studies. In addition, it was shown that the previously defined alcohol criterion value of 2.4 cm fell within the 95% CI of the mean ΔSDLP following alcohol in all nine studies that were included in the current analysis. The overall mean ΔSDLP of 2.5 cm following at a BAC of 0.5 g/L approaches the predefined cutoff value of 2.4 cm found by Louwerens et al. (1987) at the same BAC. This supports the notion that this value can and should be used as a cutoff point for determining clinically relevance of driving impairment observed for drugs other than alcohol when screened in the standardized highway driving test.

The present analysis did not reveal any difference between the magnitude of alcohol-induced impairment in males and females. In contrast, Louwerens et al. (1987) reported higher alcohol induced changes in SDLP in females as compared to males. Two explanations may be offered for this discrepancy. First, increased sensitivity for alcohol in females only became apparent at a BAC of 0.6 g/L and higher in the study by Louwerens et al. (1987). In the present studies, however, BAC values never exceeded a BAC of 0.5 g/L and thus may not have been sufficient to evoke a gender difference. Second, alcohol dosing in the study by Louwerens et al. (1987) was adjusted for body weight but not for gender differences in volume distribution (i.e., lean body mass) (Goist and Sutker 1985; Watson et al. 1980). This actually resulted in higher BACs in female participants compared to males receiving the same amount of alcohol/kilogram body weight. In contrast, more than half of the studies in the current analysis took the difference in volume distribution of alcohol between gender into account, leading to equal BACs between males and females. The present demonstration of the absence of gender specific sensitivity for alcohol effects on SDLP is also in line with a recent review of alcohol impaired driving. Martin et al. (2013) reviewed the scientific literature on alcohol-induced impairment as reported in neurocognitive, simulator, closed-course, and on-road driving studies and concluded that gender had little impact on alcohol-induced impairment at BAC levels below 1.0 g/L. Together, these data suggest that an SDLP criterion value of 2.5 cm can be reliably applied across gender to define alcohol-induced impairment at a BAC of 0.5 g/L.

Symmetry analysis confirmed the finding that alcohol significantly increases mean SDLP. It was expected that the number of individual drivers whose ΔSDLP exceeded the criterion value of 2.4 cm exceed the number of drivers whose driving actually improved by more than −2.4 cm. In the absence of any alcohol effect, the distribution of ΔSDLP for individual drivers above and below the criterion value of 2.4 and −2.4 cm was expected to be the same assuming a normal distribution of random changes. Alcohol at a BAC of 0.5 g/L was associated with 47.3% of individual drivers whose ΔSDLP exceeded the criterion value of 2.4 cm. In contrast, only a small proportion (2.2%) of drivers demonstrated a ΔSDLP that fell below the mirrored criterion of −2.4 cm. However, the cutoff point for individual performance changes in SDLP has not yet been formally validated and should therefore deserve further research.

One point that should be noted is that observed BACs during the driving test decreased over time. Mean BAC during driving (i.e., average of BAC at onset and end of the driving test) was therefore somewhat lower than the targeted BAC of 0.5 g/L at the onset of the driving test. This means that the clinically relevant cutoff point previously used in clinical trials was rather conservative and might actually be higher. Due to legal restrictions, it is unwarranted to reach a BAC higher than 0.5 g/L at the start of the driving test. Still, the current estimation of ΔSDLP at a BAC of 0.5 g/L provides a well validated and close estimate of the minimal degree of drug-induced driving impairment in the standardized highway driving test that can be associated with actual crash risk.

In conclusion, the present study showed and replicated a robust overall mean ΔSDLP of 2.5 cm during highway driving at a BAC of 0.5 g/L. These data indicate that ΔSDLP of 2.5 cm can be reliably used to determine clinical relevance of drug-induced driving impairment in the standardized highway driving test.
